# Treatment of Rats with Apocynin Has Considerable Inhibitory Effects on Arylamine *N-*Acetyltransferase Activity in the Liver

**DOI:** 10.1038/srep26906

**Published:** 2016-05-31

**Authors:** Sheena Francis, Nicola Laurieri, Chukwuemeka Nwokocha, Rupika Delgoda

**Affiliations:** 1 Natural Products Institute, University of the West Indies, Mona, Kingston 7, Jamaica; 2 Department of Pharmacology, University of Oxford, Mansfield Road, Oxford, OX1 3JA, United Kingdom; 3 Department of Basic Medical Sciences, Physiology section, University of the West Indies, Mona, Kingston 7, Jamaica

## Abstract

The effect of apocynin on the activity of arylamine *N-*acetyltransferases (NATs) in excised liver samples was examined using eighteen Sprague-Dawley rats. Three groups of six animals each were fed a normal diet alone or a treatment of 50 or 100 mg/kg/day of apocynin via gavages for eight (8) weeks. Chronic *in vivo* administration of apocynin led to significant (p < 0.001) reduction of *in vitro* liver NAT activity up to 93% as compared with untreated rats (18.80 ± 2.10 *μ*mols *p*-anisidine/min/*μ*g liver protein). *In vitro* exposure of untreated liver homogenates to apocynin led to a dose-dependent inhibition of NAT activity with IC_50_ = 0.69 ± 0.02 mM. In silico modelling of apocynin tautomers and radical species into human NAT crystal structures supported the hypothesis that thiol functionalities in NAT enzymes may be crucial in apocynin binding. The involvement of human NAT enzymes in different pathological conditions, such as cancer, has encouraged the research for selective NAT inhibitors in both humans and animal models with possible chemopreventive properties.

Apocynin, 4-hydroxyl-3-methoxyacetophenone or acetovanillone, was originally isolated from the roots of *Apocynum cannabinum* L. (Apocynacaeae) and also found in *Picrorhiza kurroa* Royle ex Benth. (Scrophulariaceae); this natural product has been traditionally used in the Aryurvedic medicine for a range of illnesses including heart and lung diseases^1^.


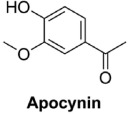


In recent years, apocynin has gained significance as a potential prodrug for multiple diseases, although its complete mechanism of action is not fully elucidated^1^. Apocynin is considered to act as an antioxidant because it prevents the activity of NAD(P)H oxidase enzyme from generating reactive oxygen species (ROS), such as superoxide anion (O_2_^−^)^2^, and thus may be useful in the treatment of a variety of illnesses which are triggered or exacerbated by an elevated inflammatory response.

Initially, apocynin was found to inhibit NAD(P)H oxidase in neutrophiles^3^, wherein its inhibitory effect appeared to be closely mediated by myeloperoxidase (MPO) enzyme^4^. The reaction of peroxidases generates radical species of apocynin, which subsequently form dimers^5^; these dimers are capable of oxidizing essential cysteine thiol groups within the sub-units of NAD(P)H oxidase^6^, thereby inhibiting the formation of the complex and its catalytic activity^7^. Nevertheless, some controversy does exist around the exclusive antioxidant role of apocynin for ROS formation processes and unfavourable pro-oxidant effects^8^. Apocynin widely seems to be useful *in vitro* and *in vivo* models for lipid peroxidation^9^, atherosclerosis^10^, kidney injuries^11^, and ischemia^12^. Moreover, apocynin shows low cytotoxicity^13^, and has chemopreventive properties^14^.

Many natural organic compounds are known to display a valuable potential for cancer-prevention in chemically induced carcinogenesis models^15^,^16^,^17^. Tumour-preventive strategies may include the use of phytochemicals for either their antioxidant properties which allow the modulation of the intracellular redox status finally leading to the apoptosis of tumour cells, or their inhibitory potency towards some metabolic pathways which activate procarcinogens^18^.

Whilst apocynin has been shown to be an inhibitor of certain isoforms of cytochrome P450 (CYP) enzymes^19^,^20^, its impact on other drug metabolizing enzymes has not been reported to date. Therefore, we investigated the effects of apocynin on the activity of arylamine *N-*acetyltransferase (NAT), which is an enzyme involved in the activation and phase-II metabolism of different carcinogenic xenobiotics via *N-*,*O-*, or *N,O-*acetylation^21^.

*NAT* gene is found in a variety of prokaryotic and eukaryotic species^21^. Chromosome 8 from the human genome contains two polymorphic *NAT* loci, *(HUMAN)NAT1* and *(HUMAN)NAT2*, which encode for two functionally distinct enzymes and have long been associated with pharmacogenetics^21^. Also a non-functional NAT pseudogene (NATP) is encountered on the same chromosome^21^.

Rodents possess three *NAT* loci in their genome and their corresponding enzyme products have conventionally been used as animal models to study human NATs^22^. In particular, (RAT)NAT2 enzyme shows a high functional homology with (HUMAN)NAT1, whereas active (RAT)NAT1 and (RAT)NAT3 have a partial analogy in relation to (HUMAN)NAT2 and NATP respectively, with rodent NAT3 having a lower cytosolic production and very little catalytic activity (Table 1, Supplementary Figure S1)^22^,^23^. Moreover, the arrangement of the three rat NAT loci on chromosome 16 appeared to be highly similar to that of the three mouse NATs on chromosome 8^22^; also, the mouse NAT enzymes (namely (MOUSE)NAT1, (MOUSE)NAT2 and (MOUSE)NAT3) were shown to *N-*acetylate similar probe substrates with an analogous catalytic efficiency as compared to (RAT)NAT1, (RAT)NAT2, and (RAT)NAT3 respectively (Table 1, Supplementary Figure S1)^22^,^24^,^25^,^26^,^27^.

Since extensive studies on human NAT genotype and phenotype have revealed a close relationship between NATs and different pathological conditions, such as cancer, looking for selective inhibitors of NAT enzymes in both humans and animal models has constituted a valuable tool to develop possible chemopreventive agents^21^. In particular, (HUMAN)NAT1 is up-regulated in several cancer types, thereby leading to cancer cell growth and resistance to chemotherapy, and has emerged as a viable candidate for drug development, which should lead to small molecule inhibitors for preclinical and clinical evaluation^28^. A few examples of phytochemical molecules were previously screened as possible inhibitors of human NATs from human liver^29^.

We report here the first study showing the inhibitory potency of apocynin against NAT activity in rat liver both *ex vivo*, following treatment with apocynin, and *in vitro*.

## Results and Discussion

### Inhibition of NAT activity in rat livers following treatment with apocynin

In this report we investigated the effects of apocynin on liver NAT activity in rats using both a system strategy which measures NAT enzymatic activity in livers excised from rats treated with apocynin and a molecular approach directly on native NAT activity in rat liver.

Previous investigations reported that rats fed 120 mg apocynin/kg showed 80% recovery of un-metabolized apocynin in the urine^30^ and a diet of apocynin 50–100 mg/kg had a low physiological impact on rats without toxic side effects^30^,^31^,^32^. Also, no gender-specific differences in rat NAT expression have been observed in the liver^33^. Thus, we initially investigated the impact of apocynin as un-metabolized natural biotic on liver NAT activity in rats fed normal diet or 50–100 mg apocynin/kg body for eight weeks. The activity of NAT enzymes in liver homogenates was measured using *p*-anisidine (pANS) which was previously shown to be a good arylamine substrate catalyzed by all active human and mouse NAT isoforms^27^.

**Figure 1:**
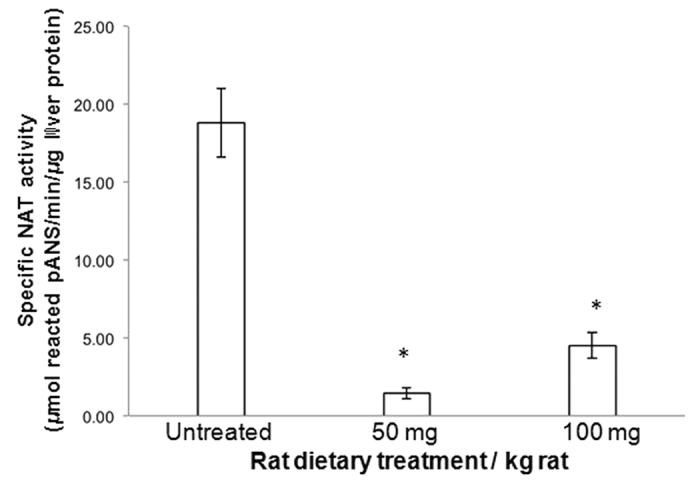
Impact of a diet containing apocynin on the activity of rat liver NATs. The NAT activity of the liver S9 fractions (3–8 *μ*g/*μ*l protein) obtained from rats fed a diet free of apocynin (untreated rats) or varying amounts of apocynin (treated rats) was determined *ex vivo* using pANS and AcCoA, as described in methods. The average value of NAT activity for the untreated group was compared with each NAT activity value for the treated groups, and statistical significance at p < 0.001 is indicated by an asterisk (*). Assays were conducted in triplicate and all values are expressed as mean ± standard deviation values.

In particular, liver NAT activity in rats fed 50 or 100 mg apocynin/kg (1.44 ± 0.35 and 4.51 ± 0.83 *μ*mols/min/*μ*g liver protein *ex vivo* respectively) appeared to be significantly reduced (p < 0.001) as compared to liver NAT activity in untreated rats (18.80 ± 2.21 *μ*mols/min/*μ*g liver protein) (Fig. 1). However, no statistically significant (p > 0.5) difference was observed between the two NAT activity measures following either of the two treatments with apocynin. Using a range of lower *in vivo* doses of apocynin would help better establish *in vivo* dose-response relationship in future pharmacokinetic studies. As far as we are aware, no report of the impact of a per os treatment with apocynin on liver NAT activity in rats without apparent undesired systemic side effects has been published previously.

In order to determine whether apocynin has also inhibitory effects against native NAT enzymes in normal rat liver, we focused on analyzing the molecular impact of apocynin on the S9 fractions prepared from liver samples of untreated rats.

**Figure 2:**
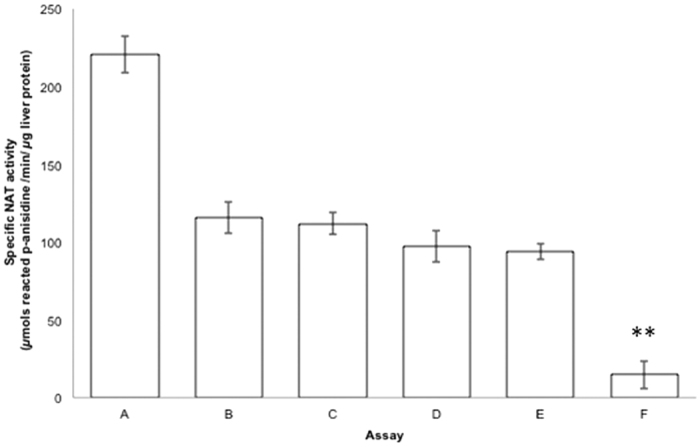
Impact of varying preincubation times of liver homogenates with apocynin and AcCoA on the inhibition of rat liver NAT activity. NAT activity in the S9 fractions (9.60 *μ*g protein) from untreated rats was determined *in vitro* as described in methods (**A**). The inhibitory potency of apocynin (0.49 mM) was tested at different assay conditions: in assays (**B–D**) apocynin was preincubated with S9 fractions for 0, 5′ or 15′ respectively; in assays E and F, S9 fractions were preincubated with AcCoA for 5′ or 15′ respectively. Appropriate enzymatic control assays were performed for each condition. Assays (**B–F**) were all statistically different from assay A (p < 0.05); ** denotes statistically significant difference (p < 0.001) between assays E and F.

Initially, apocynin or acetyl Coenzyme A (AcCoA) were allowed to preincubate with S9 liver fractions from untreated rat for either 5 or 15 minutes before starting the catalytic reaction with the other reagents. In enzymatic assays with AcCoA and pANS substrates, NAT activity was significantly reduced (p < 0.05) upon immediate addition of apocynin (assay B, Fig. 2); however, a comparable decrease of NAT activity occurred when apocynin was allowed to preincubate 5 or 15 minutes with liver homogenates prior to the addition of the two NAT substrates (assays C-D, Fig. 2). The effects of a time-dependent incubation of liver homogenates with apocynin on NAT activity apparently are not statistically dissimilar.

**Figure 3:**
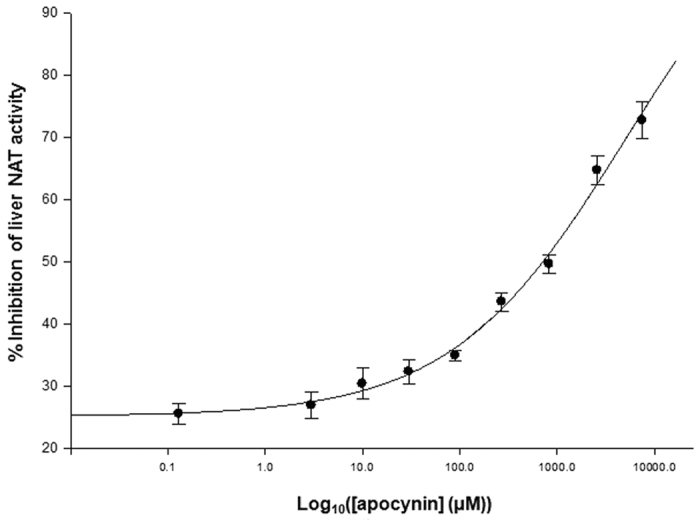
*In vitro* inhibition of NAT activity from rat liver samples by apocynin. The impact of varying concentrations of apocynin (0 and 10 mM) on the activity of rat liver NAT from S9 fractions (9.60 *μ*g protein) of untreated rat livers was investigated *in-vitro*, as described in methods. Assays were conducted in triplicate and all values are expressed as mean ± standard deviation values.

Overall, these initial results suggested a close interaction between apocynin and at least a NAT isoenzyme within rat liver homogenates, which generated inhibition of NAT activity. Additionally, apocynin showed a clear dose-dependent inhibitory potency against liver NAT activity *in vitro* with an IC_50_ value of 0.69 ± 0.02 mM (Fig. 3). IC_50_ values are known to be dependent on the amount of reactive enzyme in the assay^34^; therefore, the micromolar order of magnitude of this IC_50_ might be related to the use of liver cell lysates as source of impure NAT enzymes, instead of pure recombinant proteins. Moreover, the IC_50_ value obtained appeared to be of particular interest in comparison to previous investigations where other mammalian liver-specific enzymes, such as CYP1A2^35^ and CYP1B1^36^, were reported to be inhibited fairly weakly by apocynin in lysates from engineered prokaryotic cells over-expressing CYP enzymes^37^.

On the other hand, when AcCoA was preincubated with S9 fractions for 5 minutes, *N-*acetylation of pANS was equally lowered in the presence of apocynin (assay E, Fig. 2) as compared to assays B, C and D; this similarity looked consistent with the AcCoA preincubation times used in other enzymatic protocols using dimethylaminobenzaldehyde (DMAB) with cell lysates^38^. However, the NAT activity was doubly reduced (p < 0.001) when AcCoA was allowed to interact with S9 fractions for 15 minutes prior to the addition of apocynin and pANS (assay F, Fig. 2). This additional inhibition are likely to be related to the longer preincubation times of liver homogenates with AcCoA, since *S-*acetylated NAT intermediates can be spontaneously hydrolyzed in enzyme assays^39^, and the Coenzyme A (CoA) species formed upon AcCoA depletion can cause further inhibition of NAT activity^39^,^40^.

Enzymatic assays measuring CoA formation in NAT reaction with the colorimetric Ellman’s reagent (5,5′-dithiobis-(2-nitrobenzoic acid), DTNB) were also attempted to ascertain the extent of apocynin inhibition of NAT activity in assay F and also investigate whether apocynin can be a possible substrate of NAT enzymes^41^; however, the background noise in all spectrophotometric assay controls was very high due to the free thiol species in cell homogenates reacting with DTNB, and valuable measurements were not allowed.

### Hypothetical modes of NAT enzyme inhibition by apocynin

Amongst the studies investigating apocynin as functional enzyme inhibitor, apocynin, its radical metabolites and other polyphenolic compounds were proposed to inhibit the activity of NADPH oxidase by interacting with selective cysteine thiol groups of cytosolic sub-units thereby preventing complex formation. Two possible mechanisms were therefore suggested: a Michael conjugation between essential cysteine thiolates and the electrophilic quinone derivatives of phenols^42^,^43^, or oxidation of specific cysteine thiol groups by the phenolic radical species generated by peroxidases^7^,^44^,^45^.

In the family of mammalian NATs, cysteine residues have been well characterized in both NAT catalysis mechanism and protein structure. In particular, NAT reaction with AcCoA and arylamine substrates has been well established to follow a bi-bi ping-pong mechanism. In the first step, AcCoA binds to eukaryotic NAT protein to form a NAT-AcCoA complex, then the Cys68 thiolate within the active site is acetylated via nucleophilic addition to AcCoA, and finally CoA is delivered as first product. In the second phase, the incoming arylamine binds to the acetylated-NAT intermediate, the acetyl group is transferred to the amine substrate, and an *N-*acetylated product is finally delivered (Supplementary Figure S2)^46^,^47^. Structural investigations on Syrian hamster NAT2 helped determine this catalytic mechanism, and also highlighted the singular reactivity of the nucleophilic Cys68 thiolate towards electrophiles, such as iodo- or bromo-acetamide, in comparison with the other cysteine residues^39^. Additionally, the position and the structural functionality of the other cysteines in human NAT isoenzymes were determined via crystallography and helped visualize key intramolecular interactions in protein folding^48^.

**Figure 4:**
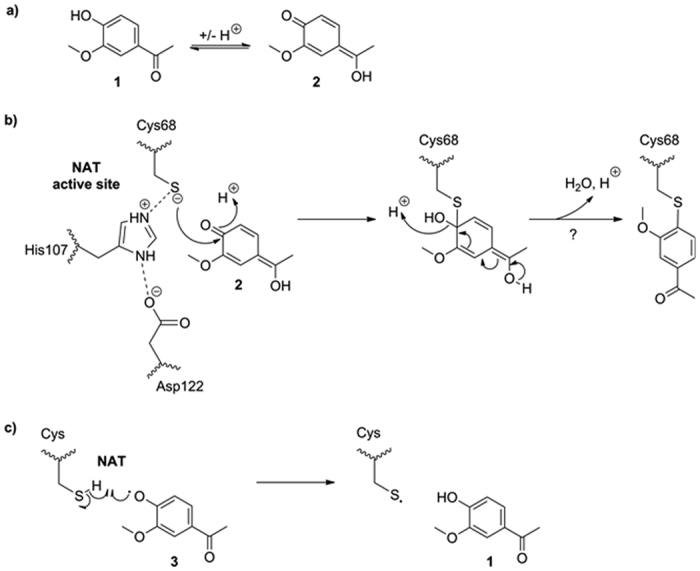
Hypothetical mechanisms of NAT inhibition by apocynin. (**a**) Tautomers of apocynin (**1**). (**b**) Possible Michael addition between Cys68 thiolate and apocynin quinonemethide tautomer (**2**) within NAT active site. Cys68, His107 and Asp122 constitute the catalytic triad in mammalian NAT enzymes^46^,^47^. (**c**) Possible interaction between exposed thiols groups of NATs and radical apocynin metabolites (**3**).

Considering the tautomeric species of apocynin (**1**) (Fig. 4a), the two possible modes of NADPH oxidase inhibition by apocynin and the role of cysteine residues in NATs, a couple of hypotheses can be proposed concerning the inhibition of NAT enzymes by apocynin in liver cell homogenates: the nucleophilic Cys68 thiolate could react with the apocynin quinonemethide tautomer (**2**) via Michael addition (Fig. 4b), possibly generating a covalent inactive adduct; alternatively, the other cysteine thiols (3 to 4) in rat NATs could be oxidized by radical apocynin metabolites (**3**), probably disrupting correct NAT folding (Fig. 4c). Also, apocynin can be hypothesized to act as a non-covalent competitive inhibitor ligand of rat NAT enzymes for its similarity in steric size or chemical shape to known arylamine NAT substrates, such as 4-aminoveratrole^27^.

### Structural simulations and biochemical validation of preliminary hypotheses

Modelling **1** and **2** or visualizing the position of oxidisable cysteines within rat NAT protein structures would be ideal to examine our hypotheses. To date, only crystal structures of human NAT enzymes are available as mammalian species^48^; nevertheless, they have constituted highly reliable templates for inhibitor studies involving rodent NATs because of their high percentages of residue identity (>70%) (Table 1, Supplementary Figure S1)^27^,^49^,^50^.

Therefore, modelling **1** and **2** into human NAT structures was attempted to explore possible distinctive intermolecular interactions between the ligand and human NAT enzymes, as models for rodent NATs. Initially, **2** was successfully docked in the crystal structures of human NATs ((HUMAN)NAT1: PDB 2PQT, (HUMAN)NAT2: PDB 2PFR)^48^, and high reliable results were obtained. In particular, the affinity energies (kcal.mol^−1^) of all the simulations with (HUMAN)NAT2 structure were lower than those with (HUMAN)NAT1, which would implicate a better affinity of **2** for (HUMAN)NAT2 than the (HUMAN)NAT1 isoform.

**Figure 5:**
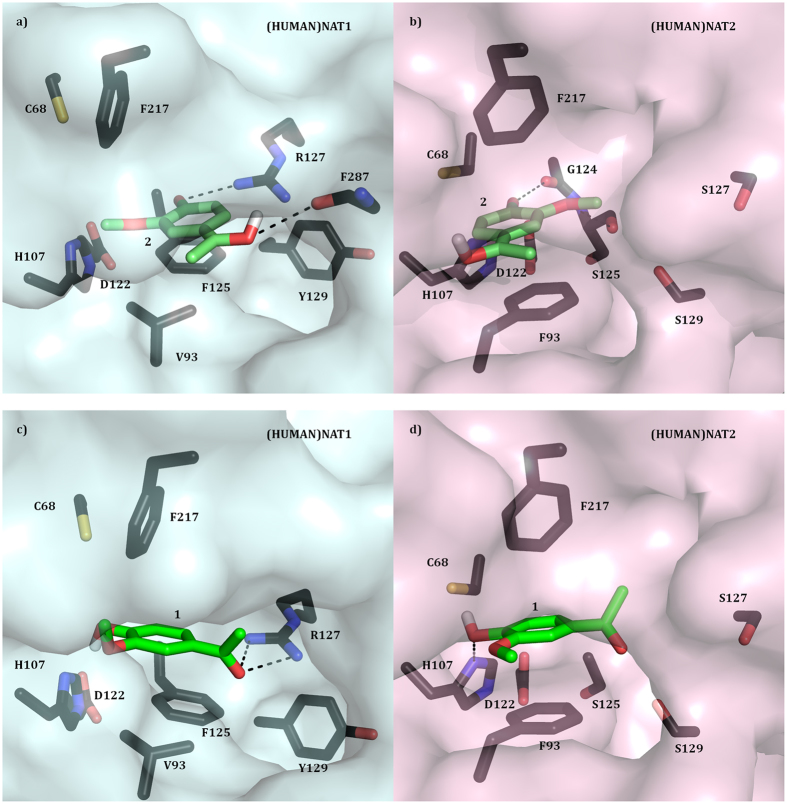
Substrate binding pockets of (HUMAN)NAT1 and (HUMAN)NAT2 with apocynin tautomers docked. **(a,b**) The active site of (HUMAN)NAT1 (**a**) and (HUMAN)NAT2 (**b**)^48^ with docked apocynin quinonemethide tautomer (**2**) is shown in surface and stick representation respectively. The residues involved in ligand binding, substrate catalysis and substrate selectivity are shown in stick representation and labelled with carbon atoms in dark blue (**a**) or dark pink (**b**), nitrogen in blue, oxygen in red, and sulphur in orange. **2** is labelled with carbon atoms in green, oxygen in red, and hydrogen in white. (**c,d**) The active site of (HUMAN)NAT1 (**c**) and (HUMAN)NAT2 (**d**)^48^ with docked **1** is shown in surface and stick representation respectively. The residues involved in ligand binding, substrate catalysis and substrate selectivity are shown in stick representation and labelled with carbon atoms in dark blue (**a**) or dark pink (**b**), nitrogen in blue, oxygen in red, and sulphur in orange. **1** is labelled with carbon atoms in green, oxygen in red, and hydrogen in white.

In one of the best simulations obtained, **2** appeared to fit within the active site of both human NATs consistent with our mechanistic hypothesis proposing a Michael addition reaction between the ligand and the enzyme (Figs 4b and 5a,b). Moreover, the compound appeared to be accommodated more internally in (HUMAN)NAT2 active site than that of (HUMAN)NAT1, which seemed coherent with the affinity energy values obtained. In details, the carbonyl carbon of the ligand was shown to point towards the thiolate functionality of C68 (4 Å (HUMAN)NAT2, 5 Å (HUMAN)NAT1), and its planar quinone ring to be sandwiched hydrophobically between the benzyl rings of two opposite phenylalanine residues (F93 in (HUMAN)NAT2, and F125 & F217 in (HUMAN)NAT1, 3–4 Å) (Fig. 5a,b). Also, the polar functionalities of **2** were proposed to interact with some polar groups within the active site via hydrogen bonds: the carbonyl O of the ligand with G124 side chain carbonyl in (HUMAN)NAT2 (3 Å) or R127 arginine guanidine in (HUMAN)NAT1 (3 Å); the methoxy O of **2** with the side chain carbonyl of S125 in (HUMAN)NAT2 (3 Å) or I106 in (HUMAN)NAT1 (3 Å) (Fig. 5a,b).

**Figure 6:**
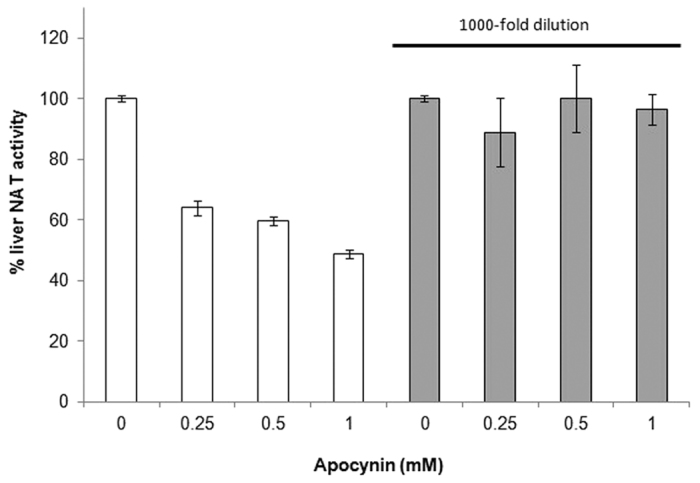
Reversible inhibition of liver NAT activity by apocynin. Apocynin inhibition of liver NAT activity appears to be reversible upon dilution. Liver lysates were incubated with varying amounts of apocynin (0–1 mM) in the assay for 30 min. The samples were subsequently diluted 1000 times, and evaluation of residue liver NAT activity was conducted using AcCoA and *p*ANS as substrates, as described in methods. Assays were conducted in triplicates.

In order to substantiate this first preliminary hypothesis, dilution experiments were performed using different concentrations of apocynin. Following preincubation of liver cell lysates with apocynin, inhibition of NAT activity by this compound was reversed upon 1000-fold dilution (Fig. 6). This would not support our first mechanistic proposition that apocynin could act as irreversible inhibitor of NAT enzymes. Nevertheless, further experiments with pure recombinant NAT proteins would be helpful.

To date, panoply of diverse sulphydryl reactive compounds has been used as irreversible inhibitors of mammalian NATs, only to elucidate the involvement of a Cys residue in the catalytic mechanism of NATs^39^,^51^,^52^,^53^,^54^,^55^ or propose hypotheses for the putative endogenous role of human NATs^56^,^57^,^58^. Other recent studies have also shown irreversible inhibition of human NAT isoforms by different heavy metals^59^,^60^, and some of the oxidizing products resulting from the oxidation metabolism of aromatic amines^61^. The eventuality that apocynin could act as covalent inhibitor of NAT enzymes would have offered some advantages in drug design, since specific covalent inhibitors are usually associated with lower doses and a longer duration of action, and avoiding resistance^62^.

Additionally, reliable docking results were successfully obtained when **1** (which is the most stable tautomer of apocynin in solution) was docked into the active site of both human NATs^63^, with a general better affinity of the ligand for (HUMAN)NAT2 for the lower affinity energies estimated. In the simulations with both human NATs, the benzene ring of the compound appeared to lay between the benzyl rings of two opposite phenylalanine residues (as shown in previous simulations with **2**) via hydrophobic π-π stacking (3 Å), and its hydroxyl tail can form a hydrogen bridge with C68 thiolate (4 Å) (Fig. 5c,d). Distinctively, the hydroxyl functionality of **1** seemed to interact also with H106 side chain imidazole nitrogen via a hydrogen bond in (HUMAN)NAT2 (Fig. 5d), whereas **1** carbonyl functionality was proposed to be locked by R127 side chain guanidine via two hydrogen bridges in (HUMAN)NAT1 (Fig. 5c).

**Figure 7:**
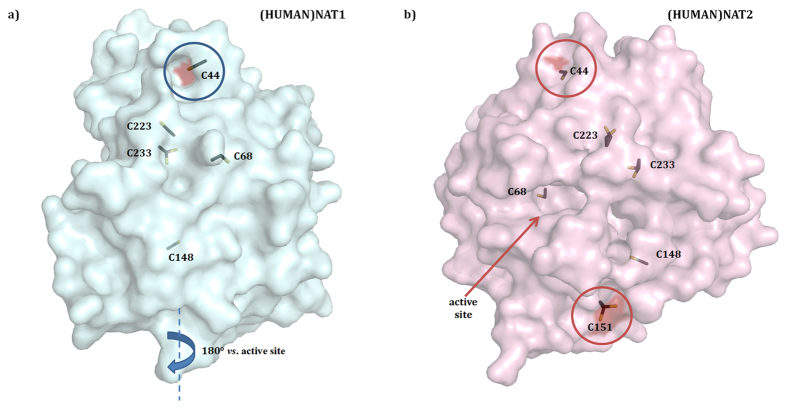
Position of cysteine residues in human NAT crystal structures. Overall surface structures of (HUMAN)NAT1 (**a**) and (HUMAN)NAT2 (**b**) are shown with cysteine side chains highlighted in stick representation as modelled in the original PDB files^48^. Sections of protein surface corresponding to outward cysteine thiols are coloured in dark red.

Thirdly, in order to evaluate the alternative hypothesis that the radical species **3** generated by MPO can inhibit NAT enzymes via oxidation of cysteine thiols (Fig. 4c), like it was earlier suggested for the cytosolic components of the NADPH oxidase complex^7^,^44^,^45^, the positions of cysteine residues in human NAT structures were examined, since most of the cysteines in mammalian NAT sequences are identical, except the additional C49 in (RAT)NAT1 (Table 2, Supplementary Figure S1). In both human NAT crystal structures, most of the cysteines are interlocked inside the protein and thus are unlikely to react with **3** for oxidation. Only C44 thiol functionality looks to point outward the protein surface (Fig. 7a,b) and can be available for oxidation by **3**: this might modify protein unfolding and subsequently inactivate the enzyme. Indeed, in both human NAT proteins, C44 thiol seems to form a hydrogen bridge with the L40 side chain carbonyl oxygen, which in turn interacts with the side chain polar groups of residues N41 (NH), I42 (NH), H43 (CO) via additional hydrogen bonds. This intricate intramolecular scaffolding appears to be consistent with the hypothesis that C44 plays a key role in correct protein folding, whose alteration upon oxidation can compromise the activity of the enzyme.

The key residues here highlighted in the formation of the complex human NAT-apocynin are analogously conserved in all rodent NAT sequences. However, additional studies about functional selectivity of rat NATs can be useful to better establish structural analogies between rat and human NAT isoenzymes and build virtual models of rat NAT proteins based on human NAT structures^27^.

In general, the complex environment of cellular lysates would demand further experiments to depict the molecular mechanism of inhibition of rat NAT activity by apocynin. Also, it is important to ascertain whether apocynin selectively affects NAT functionality in liver against a larger panel of other liver-specific enzymes, as either reactive radical or unmetabolised species, or whether indirectly down-regulates NAT gene expression levels upon oral administration. Also, studies examining NAT and myeloperoxidase activities in liver homogenates can contribute to understand the mode of inhibition of NAT by apocynin.

As *(RAT)NAT1* gene is much higher expressed in the liver than *(RAT)NAT2*^33^and has an expression pattern similar to those of (*HUMAN*)*NAT2* and (*MOUSE*)*NAT1*, which give homologous phase-II drug metabolizing enzyme products^27^,^64^, it can be suggested that most of the NAT activity encountered in rat liver homogenates can be related to (RAT)NAT1 (Table 1)^22^. It is therefore crucial to evaluate the selectivity of the inhibitory potency of apocynin against each rat NAT enzyme and establish whether apocynin might be also a potent isoenzyme-specific inhibitor against a panel of other pure mammalian NAT isoenzymes to better characterize its mode of inhibition and unravel its pharmacological potential.

As association between human NATs and different types of cancer has proposed the enzymes as possible pharmacological targets^28^,^65^, the evidence of apocynin acting as *in vivo* inhibitor of rat liver NAT activity might be of particular interest in pharmacology: importantly, apocynin has a good safety profile in all animal^30^,^31^,^32^ and human^66^,^67^ experiments reported to date, and the inactivation of NAT enzymes by apocynin can be useful to explore its potential chemopreventive effects towards arylamine carcinogenesis, since no evidence has been reported to date.

In summary, the current study shows for the first time that apocynin, a non-toxic natural organic antioxidant, constitutes a potent inhibitor of NAT activity in rat livers both *in vivo* and *in vitro* experiments. Following virtual modelling analyses, thiol functionalities in NATs are hypothesized to play an important role for apocynin binding to the enzyme. Since NAT enzymes in humans have been associated with a wide range of cancers and proposed as potential drug targets^38^,^49^,^64^, looking for possible NAT-selective inhibitors from the available range of non-toxic phytochemicals can constitute a valid approach to develop novel chemopreventive agents.

## Methods

### General Experimental Procedures

All chemical reagents were of analytical grade. Apocynin (Acetovanillone) was purchased from Sigma-Aldrich, MO, USA; purity >98%. Complete Protease Inhibitor Cocktail tablets, EDTA free, were purchased from Roche Diagnostics, GmbH, Mannheim, Germany. All other chemicals were purchased from Sigma-Aldrich, MO, USA, unless otherwise stated.

### Animal Care

The animals used were housed and cared for at the animal house at the University of the West Indies Mona. Experimental procedures on the animals were conducted after receiving ethical approval from UHWI/UWI/FMS ethics committee (protocol number AN 1 11/12). The principles of laboratory animal care were followed in accordance with specific national laws where applicable. Eighteen (18) adult male Sprague-Dawley rats of similar age (20 weeks), weighing 250–300 g, were divided into three (3) groups, with 6 rats per each group, as previously described^32^. The animals were housed in the animal house, under standard conditions of 12-h light/dark cycle. The group of untreated rats (1) was fed a normal diet consisting of rat chow and tap water ad libitum. Groups 2 and 3 were fed the normal diet together with apocynin 50 mg or 100 mg/kg/day respectively via gavages. The rats were treated for 8 weeks until they were sacrificed. We maintained an aseptic environment throughout the course of study at the best possible conditions.

### Liver Homogenate Preparation

4 g of liver tissue were dissected from each adult rat immediately after cervical dislocation and washed quickly in PBS. The tissues were minced and homogenized at 440 rpm (25% w/v) in chilled buffer (10 mM KPB, pH 7.4) containing 0.15 M KCl and 0.1 mM PMSF protease inhibitors (Roche Diagnostics, GmbH, Mannheim, Germany). The suspensions were spun in a refrigerated (4 °C) centrifuge at 9000 g for 20 min. The supernatants from each tube were labelled as S9 fractions and stored in −80 °C for further analysis.

### Protein Quantification

The amounts of proteins in all S9 fractions were determined using a modified Lowry assay kit (Thermo Scientific, Rockford IL, USA) in triplicates, as previously described^68^.

### Determination of Arylamine *N-*Acetyltransferase Activity

NAT activities in crude tissue homogenates were carried out using a modified method with pANS as substrate^40^. Routinely, reactions were conducted in triplicate at 37 °C in a final volume of 200 *μ*L which contained 100 *μ*L of an appropriately diluted S9 fraction such that NAT activity was within a linear range, with 40 *μ*L of 1 mM pANS in Tris-EDTA buffer (20 mM Tris-HCl, 10 mM NaCl, 1 mM EDTA and pH 7.5) and 40 *μ*L of 1 mM acetyl coenzyme A (AcCoA) was added to start the reaction. After 30 minutes the reaction was quenched by adding 100 *μ*L of 30% (w/v) cold trichloroacetic acid. The assay mixture was centrifuged to remove possibly precipitated protein and 200 *μ*L of 5% (w/v) DMAB were subsequently added. This allowed the spectrophotometric detection of unacetylated arylamine substrate at 450 nm using a UV-spectrophotometer (*μ*Quant universal microplate spectrophotometer, Bio-Tek Instruments, Winooski, VT, USA). pANS was chosen as arylamine substrate in all assays for its widespread reactivity with all mouse NAT enzymes homologues^27^. Enzymatic NAT activity values are expressed as *μ*mols of *N-*acetylated p-ANS/min/*μ*g of liver protein.

### Assessment of Inhibition of NAT Activity by Apocynin

For detection of inhibition of NAT activity by apocynin, minor modifications were introduced to the arylamine acetylation assay protocol used^40^. *N-*acetylation of pANS by liver homogenates was measured in the presence of apocynin following preincubation of the appropriately diluted S9 fractions (9.60 *μ*g protein) with either apocynin (0.49 mM, 0, 5 or 15 min) or AcCoA (0.20 mM, 5 or 15 min).

Varying concentrations of apocynin (0 and 7.5 mM) were also assayed *in vitro* against NAT activity from diluted liver S9 fractions (9.60 *μ*g protein) of untreated rats. Percentage inhibition was determined as the ratio of specific activity with the inhibitor related to specific activity without inhibitor. Assays were conducted in triplicate and all values are expressed as mean ± standard deviation values. To assess the nature of binding, liver lysates were incubated with varying concentrations of apocynin (0–1 mM) for 30 min prior to dilution by 1000 fold. Each sample was then assessed for residual NAT activity as earlier described.

### Data Analysis

Statistical significance of differences between average values was determined by ANOVA, the order of significance was determined using the Newman-Kuel test and the level of significance was set at p = 0.05. IC_50_ values were determined by plotting % inhibition values against log([apocynin(mM)]) and fitting the data in Sigma Plot software (version 10.0) and in the enzyme kinetics module (version 1.3), using a non-linear regression analysis model.

### Virtual Modelling Simulations

Protein sequence alignments and comparisons were performed by using ClustalW2 from the European Bioinformatics Institute^69^. Apocynin tautomers were drawn in 3D using ChemBio3D Ultra 12.0 and its ground state conformation predicted before it was docked into the catalytic pocket of the human NAT structures ((HUMAN)NAT1: PDB 2PQT, (HUMAN)NAT2: PDB 2PFR)^48^. Protein and substrate structures were defined as pdbqt files and protein-structure interactions were analyzed using Autodock Vina^70^. After adding polar hydrogen atoms to the NAT protein and defining the rotatable bonds of the ligand, the active site pocket was defined as the docking site and possible solutions were ranked according to affinity energy (lowest to highest; kcal.mol^−1^). The docking results were visualized in 3D using PyMOL^71^.

## Additional Information

**How to cite this article**: Francis, S. *et al.* Treatment of Rats with Apocynin Has Considerable Inhibitory Effects on Arylamine *N*-Acetyltransferase Activity in the Liver. *Sci. Rep.* **6**, 26906; doi: 10.1038/srep26906 (2016).

## Supplementary Material

Supplementary figures 1 and 2

## Figures and Tables

**Table 1:** Percentage Of Primary Sequence Identity of Human, Mouse, and Rat Nat Proteins against (Human)NAT1 Isoform and Probe NAT Substrate Specificities.

(HUMAN)NAT1	–							
(HUMAN)NAT2	81	–						
(MOUSE)NAT1	75	72	–					
(MOUSE)NAT2	82	75	82	–				
(MOUSE)NAT3	68	66	68	73	–			
(RAT)NAT1	76	75	92	83	69	–		
(RAT)NAT2	81	74	81	95	73	83	–	
(RAT)NAT3	69	67	75	73	88	71	72	–
	(HUMAN) NAT1	(HUMAN) NAT2	(MOUSE) NAT1	(MOUSE) NAT2	(MOUSE) NAT3	(RAT) NAT1	(RAT) NAT2	(RAT) NAT3
isoenzyme-selective substrate^a^	pABglu^b^	isoniazid	isoniazid	pABglu^b^	–	isoniazid	pABglu^b^	–

^a^Probe substrates for each NAT isoenzyme are specified where available^22^,^27^.

^b^pABglu = para-aminobenzoyl glutamate.

**Table 2:** Comparison of Cysteine Residues in Mouse and Rat NATs versus Human NAT Isoenzymes.

	Residues					
	44	68	148	151	223	233
(HUMAN)NAT1	C	C	C	R	C	C
(RAT)NAT2	C	C	A	R	C	C
(MOUSE)NAT2	C	C	A	R	C	C
(MOUSE)NAT1	C	C	A	L	C	C
(RAT)NAT1	C	C	A	R	C	C
(HUMAN)NAT2	C	C	C	C	C	C
