# Supplementary material for: Treatment of Rats with Apocynin Has Considerable Inhibitory Effects on Arylamine N-Acetyltransferase Activity in the Liver
Source: Sci Rep. 2016 May 31;6:26906. doi: 10.1038/srep26906 (PMC4886258; doi:10.1038/srep26906)
Supplement: Supplementary figures 1 and 2 [file srep26906-s1.pdf]

SUPPLEMENTARY INFORMATION

Treatment of Rats with Apocynin Has Considerable Inhibitory Effects on Arylamine *N*-Acetyltransferase Activity in the Liver

Sheena Francis, Nicola Laurieri,Chukwuemeka Nwokocha, Rupika Delgoda*

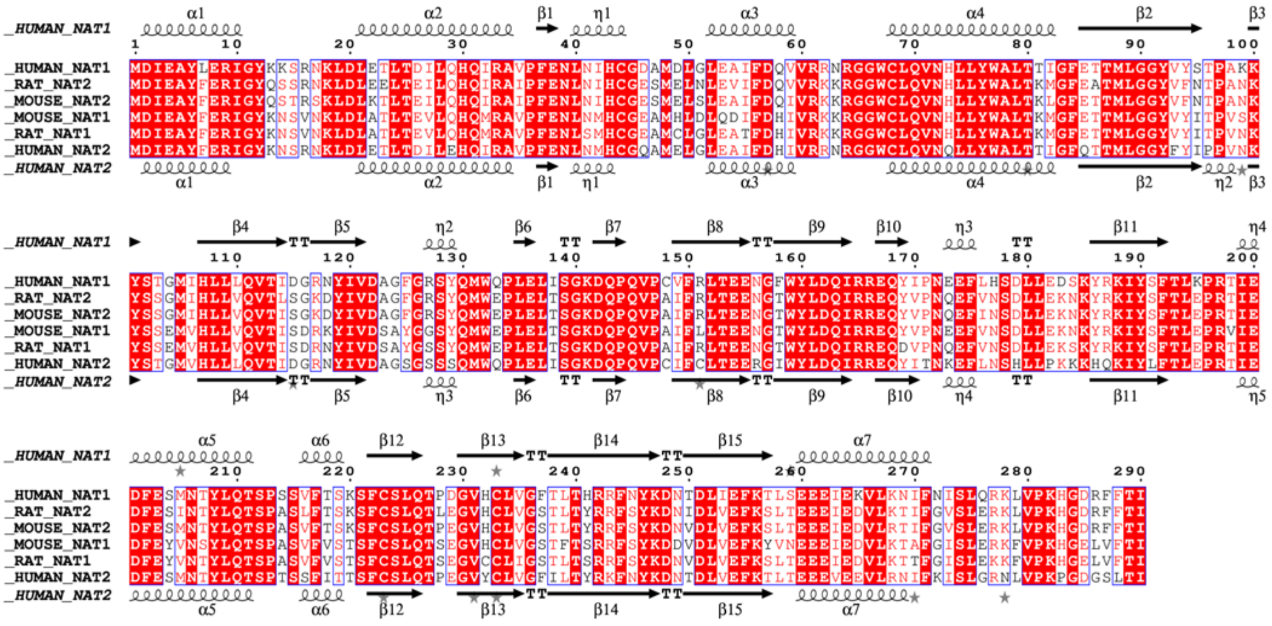

Supplementary Figure S1. Multiple sequence alignment of selected NAT proteins. Human, murine and rat NAT proteins are aligned. Similar amino acids are highlighted in blue boxes; completely conserved residues are indicated by white lettering on a red background. The secondary structures are adapted from (HUMAN)NAT1 (top, PDB 2PQT) and (HUMAN)NAT1 (bottom, PDB 2PQT) crystal structures.[1] Alignment was generated using Clustal W.[2] Figure was prepared using ESPript 2.2.[3]

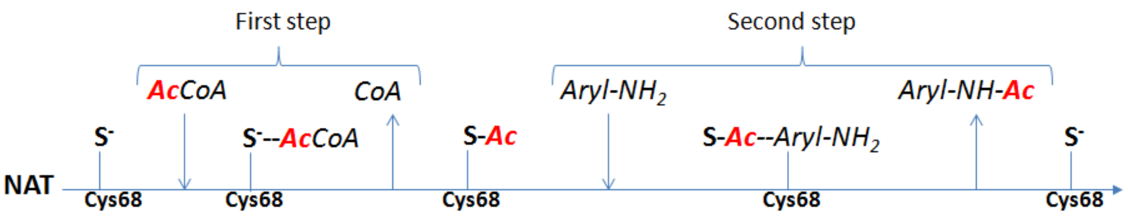

Supplementary Figure S2. Bi-bi ping-pong mechanism of NAT reaction. The bi-bi ping-pong mechanism is shown with AcCoA as acetyl donor. The residue numbering is referred to mammalian NAT sequences. The acetyl (Ac) transfer from Cys68 to the substrate occurs following nucleophilic attack of the *N*-acetylated intermediate by arylamine (Aryl-NH_2).

1 Wu, H. *et al.* Structural basis of substrate-binding specificity of human arylamine N-acetyltransferases. *J Biol Chem* **282**, 30189-30197 (2007).

2 Thompson, J. D., Higgins, D. G. & Gibson, T. J. CLUSTAL W: improving the sensitivity of progressive multiple sequence alignment through sequence weighting, position-specific gap penalties and weight matrix choice. *Nucleic Acids Res.* **22**, 4673-4680 (1994).

3 Gouet, P., Courcelle, E., Stuart, D. I. & Metoz, F. ESPript: multiple sequence alignments in PostScript. *Bioinformatics* **15**, 305-308 (1999).
